# Adaptive best subset selection algorithm and genetic algorithm aided ensemble learning method identified a robust severity score of COVID‐19 patients

**DOI:** 10.1002/imt2.126

**Published:** 2023-07-04

**Authors:** Weikaixin Kong, Jie Zhu, Suzhen Bi, Liting Huang, Peng Wu, Su‐Jie Zhu

**Affiliations:** ^1^ Institute for Molecular Medicine Finland (FIMM), HiLIFE University of Helsinki Helsinki Finland; ^2^ Institute of Translational Medicine, The Affiliated Hospital of Qingdao University, College of Medicine Qingdao University Qingdao China; ^3^ Cancer Biology Research Center (Key Laboratory of the Ministry of Education), Tongji Medical College, Tongji Hospital Huazhong University of Science and Technology Wuhan China; ^4^ Department of Gynecologic Oncology, Tongji Hospital, Tongji Medical College Huazhong University of Science and Technology Wuhan China

## Abstract

We used an integrated ensemble learning method to build a stable prediction model for severity in COVID‐19 patients, which was validated in multicenter cohorts.

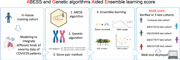

## INTRODUCTION

The COVID‐19 pandemic has caused significant damage and led to more than one million deaths in the United States and six million deaths worldwide [1], mainly due to the development of acute respiratory distress syndrome (ARDS). During the period of COVID‐19 spread, medical resources have been subjected to tremendous pressure and hospitals which has caused saturation of our health systems. Few therapeutic interventions authorized by the US Food and Drug Administration are available, but vaccine deployment has been slow in many parts of the world. On January 6, 2023, the National Health Commission and the State Administration of Traditional Chinese Medicine issued the “COVID‐19 Diagnosis and Treatment Plan (Trial Version 10, http://www.gov.cn/zhengce/zhengceku/2023-01/06/content_5735343.htm).” The clinical symptoms of COVID‐19 are divided into mild, moderate, severe, and critical. This categorization technique can be beneficial in enhancing doctors' abilities to diagnose and treat patients with COVID‐19 and further aid scientists in their comprehension of the disease's development. However, it also has its shortcomings. For example, the varying symptoms that patients display at different stages can complicate the definitive categorization process. Furthermore, this method may be tailored specifically to the disease's prevalence within specific regions or demographics, thereby limiting its universal applicability. Moreover, the batch effect of patients in different hospitals and the differences in equipment may affect the accuracy of patient stratification. Hence, it is important to develop a comprehensive and robust model to predict the potential clinical severity of SARS‐CoV‐2 infection to achieve a reasonable allocation of medical resources.

It is a good way to use ensemble learning methods to set up a robust model since ensemble learning methods have exhibited high accuracy when compared to the single machine learning method in many biological prediction tasks [2, 3, 4]. The accuracy and robustness of the ensemble model are often challenged by the feature engineering process and basic learner diversity [5]. To establish an effective ensemble learning model to predict the severity state of COVID‐19 patients, we used an Adaptive best subset selection algorithm [6] and a Genetic algorithm [7] Aided Ensemble learning score (AGAE score) for outcome state prediction of patients with COVID‐19 in this study. It is noted that the gene‐pairing method was used to eliminate the possible batch effects caused by the substantial differences in the heterogeneous patient cohorts and transcriptomic data distributions, while the Adaptive BEst Subset Selection (ABESS) algorithm and genetic algorithm were used to select the best features and basic learners, respectively. Our web tool of AGAE score (https://kwkxbioinfor.shinyapps.io/COVID19/) provided a predictive value for the degree of severity in COVID‐19 patients.

## RESULTS

### The overall design of the AGAE score

**Figure 1:**
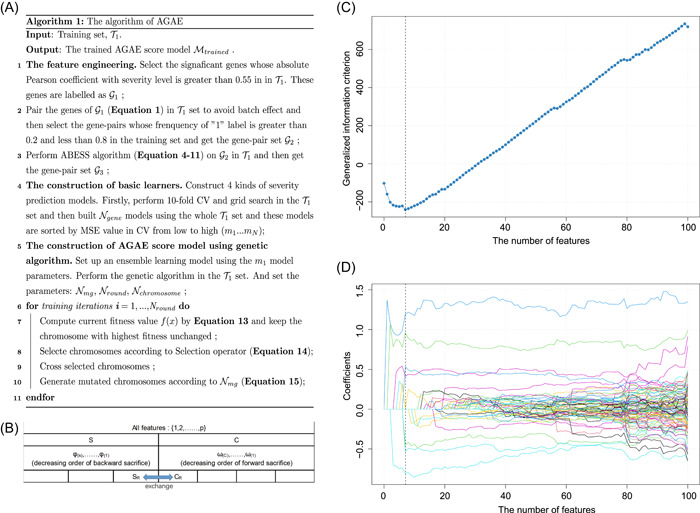
The pseudocode of AGAE score and the results of ABESS algorithm. (A) The algorithm of AGAE score. (B) The illustration of the ABESS algorithm. (C) The change in generalized information criterion (GIC) when selecting features using the ABESS algorithm. (D) The coefficients of features when selecting features.

Currently, since there are still some inconsistencies in the severity data types of COVID‐19 among different hospitals or sources [8], we aimed to establish a consistent severity score for COVID‐19 patients with high accuracy and robustness. To achieve this, we proposed an AGAE score. Specifically, the gene‐pairing method was used to eliminate the batch effect leading to improving the robustness of the model, while the ABESS algorithm was performed to select the best features as input data to train the model. Furthermore, the genetic algorithm was applied to find the best basic learner combination in the ensemble model automatically. Taken together, the overall design of the AGAE score is shown in Figure S1 and Figure 1A.

### The cohorts collected in this study

This study included five cohorts, and the in‐house data was divided into the training set and internal test set, as shown in Table S1. The training set was used to construct the AGAE score and the other sets were used as test sets to evaluate the effectiveness and robustness of the AGAE score.

### Feature selection and engineering

To find genes related to the severity state in COVID‐19 patients, we first used the Pearson coefficient between the gene expression levels and severity data (“Asymptomatic” labeled as 1, “Mild” labeled as 2, “Severe” labeled as 3, and “Critical” labeled as 4) in the training set to evaluate the importance of genes. There were 251 genes with an absolute coefficient greater than 0.55 (Figure 1) and these genes were used to perform gene‐pairing (Supporting Information: materials, Equation 1). Then, 5925 gene pairs whose frequency of the “1” label was greater than 20% and less than 80% were used as input data to perform the ABESS algorithm. In the ABESS algorithm (Figure 1C,D), when we only used seven gene pairs to predict the severity levels in the training set, the model can achieve the least generalized information criterion value [9] (Figure 1C), with the coefficients of these seven gene‐pairs shown in the Figure 1D. These seven gene pairs were constructed by 14 genes as shown in Table S2. Consequently, these seven gene pairs were regarded as optimized features to set up basic learners in the following research (Figure S1).

### Construction of 50 basic learners

To make an optimum performance of the prediction model, we performed four kinds of machine learning regression algorithms to build the basic learners, including elastic net (ERN), random forest (RF), support vector machine (SVM), and K–nearest neighbors (KNN). Considering the parameters' influence, we first performed a 10‐CV and grid search in the training set and then found ERN algorithms steadily contribute to lower mean square error (MSE) value when compared with other algorithms (Table S3), resulting in its best performance with *ɑ* = 1.0. In the end, using the parameters shown in Table S3, we identified 50 basic learners (m1–m50) to do further research.

### Construction of AGAE score

Using the stacking strategy, the predicted values of basic learners were used as input data to construct an ensemble model, namely the AGAE score. In the ensemble learning model, the diversity and accuracy of the basic learner played important roles in the performance of the final ensemble model. Given that the researchers needed to achieve this manually in initial studies [10], in this research, we used a genetic algorithm to optimize basic learner combinations to achieve higher accuracy in the ensemble model automatically, as the fitness function was shown in Supporting Information: materials, Equation (13). The fitness value decreased when the genetic algorithm was performed in the training set (Figure S2). In the fifth round of optimization, we found that the model reached convergence as well as the fitness minimized (Figure S2). At last, there were four models included in the AGAE score, namely m3, m21, m30, and m32. Among these four basic learners, m3 used the ERN algorithm, while m21 and m32 used the KNN algorithm, with m30 using the RF algorithm. To sum up, these results highlighted high diversity in the types of algorithms in selected basic learners, which proved the automation of genetic algorithms. Finally, the AGAE score was constructed in the training set based on these four basic learners' predicted values as input data, and the methods and parameters of the ensemble learning model are consistent with m1 (ERN model, *α* = 1.0). Details can be seen in the method part (Supporting Information: materials).

### Evaluation of AGAE score

**Figure 2:**
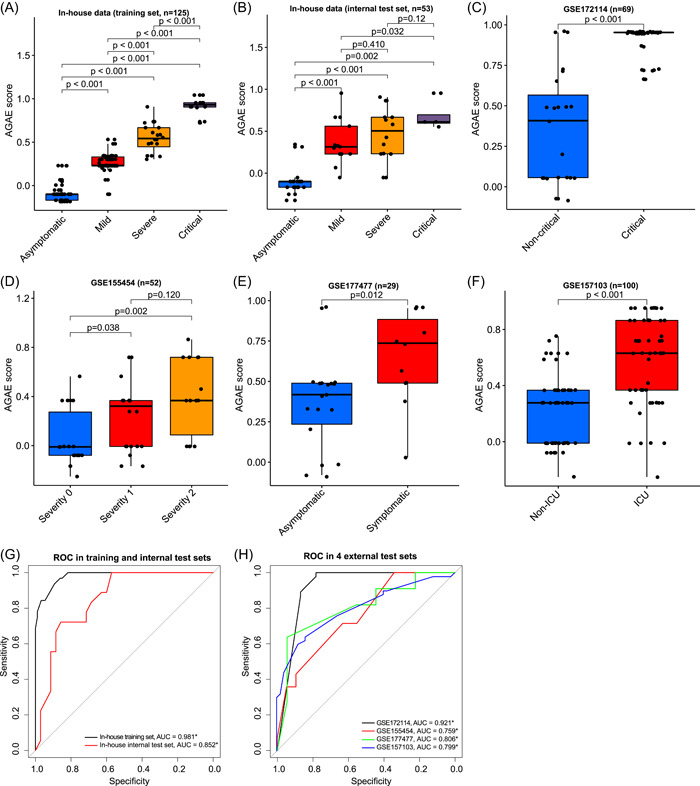
The performance of AGAE score in the training set and five test sets. (A–F) The comparison of AGAE scores in the different severity (Wilcoxon test). (A) In‐house data (training set), (B) in‐house data (internal test set), (C) GSE172114, (D) GSE155454, (E) GSE177477, (F) GSE157103. (G and H) The ROC curves of AGAE (300‐turn permutation test). The samples were divided with different AGAE score thresholds to draw the ROC curves of severity data. (G) The training set and test set (in‐house data). (H) Four external test sets.

To further investigate the severity prediction performance of the AGAE score, we applied it to the training cohort and five independent COVID‐19 patient cohorts (four external cohorts and one internal test cohort). Notably, the AGAE score exhibited a significant difference among different severity groups across the training and five test groups (Figure 2A–F), and the patients with higher AGAE scores tended to have worse severity states. We further performed area under the receiver operating characteristic (ROC) curve (ROC‐AUC) analysis for AGAE score across all COVID‐19 cohorts (Figure 2G,H). Since ROC curves can only be used in the binary classification task, some severity groups in certain cohorts were combined to construct binary groups (see method part, Supporting Information: materials). As expected, the AGAE score achieved significant ROC‐AUCs across all the COVID‐19 cohorts (Figure 2G,H, 300‐turn permutation test), with the least and average ROC‐AUCs in test sets standing at 0.759 and 0.827, respectively (Figure 2G,H), which proved the effectiveness and robustness of AGAE score. Then, we also visualize the distribution of AGAE scores among different cohorts (Figure S3). The distribution of AGAE scores in these cohorts is relatively consistent, but in the GSE172114 cohort, most of the AGAE scores have larger values (Figure S3C). It can be seen from Figure 2C that in the GSE172114 cohort, many patients belong to the “Critical” category, which is very consistent with the distribution of AGAE scores in GSE172114. The above results prove the rationality of the AGAE score.

To further demonstrate AGAE score effectiveness, we used four basic learners as well as the m6A score established by Qiu et al. [11] as baseline models and found that AGAE score had higher ROC‐AUCs (*p* < 0.05, paired *t*‐test, Table S4) significantly except the m3 model. Although the *p*‐value was not significant (0.076) in the comparison with the m3 model (Table S4), the ROC‐AUCs of AGAE score were still higher than that of m3 among all test sets. Then, COVID‐19 Severity Index [12] was also used to be compared with the AGAE score. Only the in‐house cohorts and GSE157103 cohort had detailed clinical information, based on which COVID‐19 Severity Index can be calculated, so the comparison was performed on these cohorts. The result was shown in Figure S4A,B. The ROC‐AUCs of the AGAE score were higher than those of the Severity Index in both the in‐house test cohort and the GSE157103 cohort, which proved the effect and robustness of the AGAE score.

**Figure 3:**
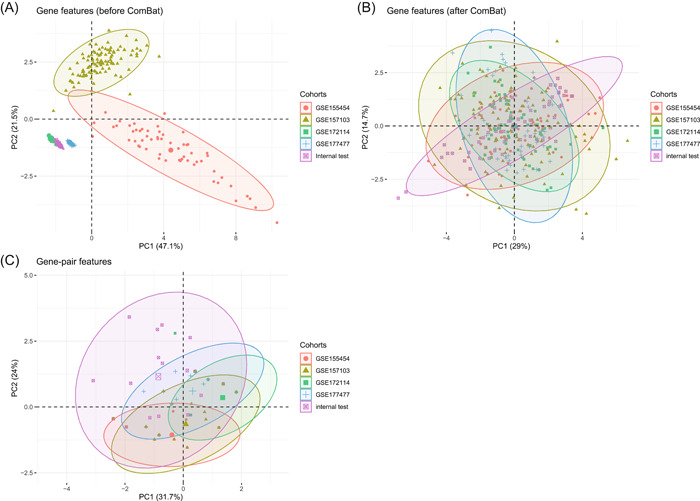
Comparison of different methods to eliminate batch effect. (A) The PCA analysis of unpaired gene features in five test cohorts. (B) The PCA analysis of unpaired gene features in five test cohorts after using the ComBat function. (C) The PCA analysis of gene‐pair features in five test cohorts. PCA, principal component analysis.

To verify whether the gene‐pairing method, ABESS algorithm, and genetic algorithm contribute to the improvement of accuracy in AGAE score effectively, we further performed PCA analysis and ablation experiments. When trying to combine five test cohorts, we found that the distributions of gene features chosen to construct the AGAE score were distinct among various COVID‐19 cohorts (Figure 3A), displaying the batch effect and heterogeneity in these sets. Although the ComBat function was able to be applied to eliminate the batch effect (Figure 3B) to obtain similar distributions, the original expression data was changed in that situation, which may introduce noise to subsequent data analysis. It is noted that using gene‐pair features without the ComBat, the distributions of these test sets were similar (Figure 3C), highlighting that the gene‐pairing method not only reduces batch effect across different sets but also keeps the original expression data information unchanged. Another interesting phenomenon is that there are fewer points in Figure 3C, compared with Figure 3A,B. Each point represents a sample, which means that there are many patients with the same features of the gene pairs.

We further explored the performance of the gene‐pairing method by ablation experiments, which referred to first deleting the gene‐pairing part and then using the gene features directly to set up the AGAE score. Without the gene‐pairing method, the ROC‐AUCs of the AGAE score were significantly lower than those of the original AGAE score (*p* < 0.05, paired *t*‐test, Table S5). As we expected, the ROC‐AUC in GSE155454 was only 0.523 (Figure S4C,D) which proved the prediction model useless in this test set (*p* > 0.05, 300‐turn permutation test), while AUC‐ROCs of other test sets were significant, demonstrating the decrease of the robustness of AGAE score. In the next step, to verify the effectiveness of the ABESS algorithm, we deleted this part during the construction process of the AGAE score and used the traditional LASSO method instead. Using the LASSO method, we obtained 41 gene features from 251 genes (Figure S5) which can achieve the least MSE value (Figure S5A), and these 41 genes were used to set up basic learners and then set up the new AGAE score. However, as compared with the original AGAE score, the new AGAE score without the ABESS algorithm had lower ROC‐AUCs significantly (*p* < 0.05, paired *t*‐test, Table S5 and Figure S6A,B), emphasizing the effectiveness of the ABESS algorithm. As for the ablation experiment for the genetic algorithm, we deleted the genetic algorithm part and set up the AGAE score directly using all 50 basic learners. When compared to the original AGAE score, the AGAE score without a genetic algorithm displayed lower ROC‐AUCs (*p* < 0.05, paired *t*‐test, Table S5 and Figure S6C,D), which verified the necessity of a genetic algorithm in the AGAE score.

Together, the above results suggested that it is necessary to employ the gene‐pairing method, ABESS algorithm, and genetic algorithm for improving the robustness and accuracy of the ensemble model.

In addition, the clinical features can also serve as an important index to predict the severity of COVID‐19, such as mechanical ventilation, age, C‐reactive protein (CRP), and so on. We, therefore, analyzed the potential relationship between AGAE score and clinical features in the GSE157103 cohort. There was no difference in AGAE scores between the male and female patients with COVID‐19 (Figure S7A), while the patients who were treated with mechanical ventilation had higher AGAE scores than others (Figure S7B) and this was reasonable because the patients with mechanical ventilation tend to be patients with a higher degree of severity and complications. It was noted that AGAE score could be positively correlated with APACHE‐II score (*R* = 0.49, *p* < 0.001), SOFA score (*R* = 0.50, *p* < 0.001), age (*R* = 0.23, *p* = 0.025), ferritin (*R* = 0.24, *p* = 0.019), d‐dimer (*R* = 0.46, *p* < 0.001) and CRP (*R* = 0.27, *p* = 0.009), and negatively correlated with ventilator‐free days (*R* = −0.47, *p* < 0.001) (Figure S7C–I). Among these clinical characteristics, the APACHE‐II score [13] can be used to evaluate the severity of the disease, whereas the SOFA score [14] can be adopted to evaluate the degree of organ failure.

It showed a similar trend between the above two kinds of scores and our AGAE score, which indicated that the AGAE score can be an effective index of not only disease severity but also organ failure. Moreover, we observed that the increase of ferritin, d‐dimer, and CRP was consistent with a high AGAE score, given that these three characteristics were regarded as features of cytokine storm and excessive inflammation [15], which may cause a higher mortality rate, so this result is reasonable and consistent.

All of these observations showed that the AGAE score can serve as a powerful index to predict the disease severity in patients with COVID‐19. To make the AGAE score widely used by other researchers, we deployed it as a web tool (https://kwkxbioinfor.shinyapps.io/COVID19/), in which the users only need to input the expression data of 14 genes for a specific patient to obtain the AGAE score.

### Single sample gene set enrichment analysis (ssGSEA) and gene set variation analysis (GSVA) in low and high AGAE score groups

Given that patients with severe COVID‐19 exhibit substantial immune changes including lymphopenia [16, 17, 18], we also explored the components of the different lymphocytes between the low AGAE score group and high AGAE score group in the training set (divided by median value). It was noted that high AGAE score group patients had lower activated B cells, activated CD8 T cells, activated dendritic cells, and MDSC cells (Figure S8A). The previous study has shown that the severe/critical patients' antigen processing and presentation ability was significantly reduced [19], which was consistent with our result (Figure S8A).

To better illustrate the biological behaviors between the two different groups, we performed GSVA to compare the distinct KEGG signaling pathways between the low AGAE score and high AGAE score groups. It was observed that the high AGAE score group displayed a decrease in the T cell receptor signaling pathway, B cell receptor signaling pathway, and Leishmania infection pathway (Figure S8B), which was consistent with the above ssGSEA result and the report that COVID‐19 was associated with combining infection state [20]. The above results showed that the high AGAE score group contained a relatively lesser number of immune cells that may hamper the ability to mount an effective antiviral response. Then, to further explore the relationship between the 14 genes in the AGAE score and the immune process, we used the ESTIMATE algorithm [21] to calculate ImmuneScore in the training set. ImmuneScore can represent the content of immune cells and the degree of immune response. Correlation analysis (Spearman coefficient) is shown in Figure S9. These 14 genes were all significantly correlated with the immune response (*p* < 0.001), among which 10 genes were positively correlated and four genes were negatively correlated. This indicates that the AGAE score is mainly a score based on changes in the immune function of COVID‐19 patients, which is consistent with the above ssGSEA and GSVA results.

## DISCUSSION

In this study, we first established an ensemble learning method to predict disease severity for COVID‐19 patients, called the AGAE score. Further experiments emphasized the effectiveness and superiority of the AGAE score compared to other baseline models or m6A scores as well as the necessity of gene‐pairing, ABESS algorithm, and genetic algorithm during the process of construction of the AGAE score. Specifically, the gene‐pairing method can eliminate the batch effect, while the usage of the ABESS algorithm and genetic algorithm contributes to improving the accuracy of the model. This combined ensemble learning method can be used in either COVID‐19 or any disease with enough expression data and key clinical response data, such as the prognosis prediction in cancer.

The AGAE score is a kind of seven‐gene‐pair signature, which contained 14 genes. Among these 14 genes (Table S2), some of them are reported to be closely related to COVID‐19. RNF187 has been shown to be a biomarker in children with COVID‐19 [22]; NCR3 is significantly associated with the decreased proportion of CD8+ T and T helper 2 cells in the peripheral circulation in COVID‐19 patients [23]; TEX2 can predict the ventilator‐free days of patients with COVID‐19 [24]; ITPKB is a hub gene shared by Alzheimer's disease and COVID‐19 [25]; TUBG1 is involved in the immune interaction of COVID‐19 and lymphoma [26]; RPL14 is a significantly upregulated gene in COVID‐19 patients [27]. Besides the above genes, other unreported genes in the AGAE score may also have research significance.

Although the AGAE score has achieved good performance, some limitations still need to be in‐depth considered. First, as the use of the AGAE score requires blood draws from the patient, more safety issues should be further concerned. If the model could be built on noninvasive data, such as urine samples [28], it will help to improve the clinical application value of the model. Second, compared with basic learners, the improvement of AGAE score is still limited, this may be due to the limited size of the training set. With the amount of data accumulating, other machine learning methods can be applied to improve the AGAE score, such as transfer learning. In addition, the severity of COVID‐19 patients is also firmly related to virus subtypes, so in the following research, we will also try to collect virus subtype information of COVID‐19 patients, to establish virus‐subtype‐specific severity prediction models to improve precision medicine in clinical practice.

## CONCLUSIONS

In this study, we proposed an AGAE score. It exhibited a powerful ability in the severity prediction of COVID‐19 patients. Compared with the other five baseline models, the AGAE score achieved the highest average ROC‐AUC (0.827) in five independent test sets. Our ablation experiments proved that the gene‐pairing method, ABESS algorithm, and genetic algorithm applied can eliminate the batch effect, and improve model accuracy and effectiveness, respectively. Furthermore, an easy‐to‐use web tool for AGAE score (https://kwkxbioinfor.shinyapps.io/COVID19/) was available.

## AUTHOR CONTRIBUTIONS

Sujie Zhu, Weikaixin Kong, and Jie Zhu interpreted this study and wrote the manuscript. Weikaixin Kong advised on aspects of study design, and, together with Sujie Zhu and Jie Zhu, performed data collection, curation, and analysis. Suzhen Bi, Liting Huang, and Peng Wu verified the underlying data. All authors contributed to the article and approved the submitted version.

## CONFLICT OF INTEREST STATEMENT

The authors declare no conflict of interest.

## ETHICS STATEMENT

This was a retrospective study and all ethics approvals had been obtained in other researchers' studies. The cohort data can be obtained in public databases, and the access number can be found in Table S1.

## Supporting information

Supporting information.

Supporting information.

## Data Availability

The codes of the AGAE score are available at https://github.com/kwkx123/AGAE; the web‐tool is available at https://kwkxbioinfor.shinyapps.io/COVID19/. The cohort data can be obtained in public databases, and the access number can be found in Table S1. Supporting Information: materials (figures, tables, scripts, graphical abstract, slides, videos, Chinese translated version, and updated materials) may be found in the online DOI or iMeta Science http://www.imeta.science/.
