# Supplementary material for: Adaptive best subset selection algorithm and genetic algorithm aided ensemble learning method identified a robust severity score of COVID‐19 patients
Source: Imeta. 2023 Jul 4;2(3):e126. doi: 10.1002/imt2.126 (PMC10989835; doi:10.1002/imt2.126)
Supplement: Supplementary file 1 — Supporting information. [file IMT2-2-e126-s002.docx]

## Supporting information to:

**Adaptive Best Subset Selection Algorithm and Genetic**

**Algorithm aided Ensemble Learning Method Identified a Robust Severity Score of COVID-19 Patients**

Running title: AGAE score：a robust severity score of COVID-19 patients

Weikaixin Kong^1#*^, Jie Zhu^1#^, Suzhen Bi^2^, Liting Huang^2^, Peng Wu^3,4*^ & Su-Jie Zhu^2 *^,

^1^ Institute for Molecular Medicine Finland (FIMM), HiLIFE, University of Helsinki, Helsinki, Finland

^2^ Institute of Translational Medicine, The Affiliated Hospital of Qingdao University, College of Medicine, Qingdao University, Qingdao, China

^3^ Cancer Biology Research Center (Key Laboratory of the Ministry of Education), Tongji Medical College, Tongji Hospital, Huazhong University of Science and Technology, Wuhan, China.

^4^ Department of Gynecologic Oncology, Tongji Hospital, Tongji Medical College, Huazhong University of Science and Technology, Wuhan, China.

# These authors contributed equally to this work

*Correspondence:

zhusujie@qdu.edu.cn (Su-Jie Zhu); [pengwu8626@tjh.tjmu.edu.cn](mailto:pengwu8626@tjh.tjmu.edu.cn) (Peng Wu); kong.weikaixin@helsinki.fi (Weikaixin Kong)

## Methods and materials

### The COVID-19 cohorts used in this research

We collected 5 COVID-19 patient cohorts (Supplementary Table1) which contained both expression data and severity data, including the in-house cohort (n=178), GSE172114 (n=69)[1], GSE155454 (n=52), GSE177477 (n=29)[2], GSE157103 (n=100)[3]. The in-house cohort contained 178 patients and the RNA sequencing process was described in our initial publication[4], and then this in-house data was divided into the training set (n=125) and internal test set (n=53) randomly, while the other 4 cohorts were also used as independent test sets. The expression data of GSE177477 was microarray data, whereas other cohorts were all RNA-seq data. The detailed clinical information and the types of severity data in these 5 cohorts were described in Supplementary Table 1. The data of GSE172114, GSE155454, GSE177477 and GSE157103 were downloaded from the GEO database (<https://www.ncbi.nlm.nih.gov/geo/>).

### The framework of the AGAE score

The Adaptive best subset selection algorithm and Genetic algorithm Aided Ensemble learning model (AGAE score) was established to predict the severity for COVID-19 patients, as shown in Supplementary Figure 1 and Figure1A. We firstly performed feature selection and engineering, including the gene-pairing process and ABESS algorithm, and then used the genetic algorithm to find the best basic learner combination in the ensemble model, which enabled us to construct the AGAE score, before this model was used to perform further analysis.

### Feature selection and engineering

The whole feature processing was performed in the training set. To get key genes related to the severity state of COVID-19 patients, we firstly used the Pearson coefficient to select genes, and there were 251 genes whose absolute value of coefficient was greater than 0.5 (Supplementary Figure 1). To avoid batch effect influence, these genes were paired to get gene-pair features (31375 gene-pairs) based on equation 1, followed that the 5925 gene-pairs whose frequency of “1” was greater than 0.2 and less than 0.8 were left aimed to obtain gene-pairs which contained enough difference across different patients. This ratio is chosen according to existing research[5, 6].

$\text{Gene A|Gene B}=\left\{ \begin{aligned} 1, Expression\left( A \right)>Expression\left( B \right) \\ 0, Expression\left( A \right)\leq Expression\left( B \right) \end{aligned} \right.$ (1)

Next, the ABESS algorithm was used to select the best features for 5925 gene-pairs, and detailed information about the ABESS algorithm can be found in the next section.

### The ABESS algorithm

It was a regression task for the establishment of severity score (Equation 2, $\beta$ is the feature vector and $\varepsilon$ is the constant term).

$y=x^{T}\beta+\varepsilon$ (2)

In the feature selection process of the regression task, the Best Subset Selection algorithm (ABESS) proposed by Zhu et al.[7] has exhibited better performance on the data with high-dimensional features when compared to traditional LASSO regression[8] (Equation 3, p is the number of all features and s is a constant term), noted that this algorithm was based on the splicing technique.

$\arg\min_{\beta} \sum_{i=1}^{N} {(y_{i}-\beta_{0}-\sum_{j=1}^{p} x_{ij}\beta_{ij})}^{2}$ subject to: $\left\| \beta\right\|_{1}\leq s and \left\| \beta\right\|_{1}=\sum_{j=1}^{p} \left| \beta_{j} \right|$ (3)

In the ABESS algorithm, our defined subset of selected features is $S=\{j:\beta_{j}\neq0\}$, and the size was s, the complement set of features was $U=\left\{ 1,\ldots,p \right\}\backslash S$. When a fixed S was given, the least squares estimate can be got according to equation 4.

$\hat{\beta}=\arg\min_{\beta_{U}=0} \sum_{i=1}^{N} {(y_{i}-\beta_{0}-x_{i}^{T}\beta)}^{2}$ (4)

Then as for the feature j in set S, the feature’s backward sacrifice $\varphi_{j}$ was defined as the increase of bias when excluding the feature j in set S:

$\varphi_{j}=\sum_{i=1}^{N} {(y_{i}-\hat{\beta}_{0}-x_{i}^{T}\hat{\beta}+x_{ij}\hat{\beta_{j}})}^{2}-\sum_{i=1}^{N} {(y_{i}-\hat{\beta}_{0}-x_{i}^{T}\hat{\beta})}^{2}$ (5)

As for the feature j in set C, the feature’s forward sacrifice $\omega_{j}$ was defined as the decrease of bias when including the feature j in set S:

$\omega_{j}=\sum_{i=1}^{N} {(y_{i}-\hat{\beta}_{0}-x_{i}^{T}\hat{\beta})}^{2}-\sum_{i=1}^{N} {(y_{i}-\hat{\beta_{0}}-x_{i}^{T}\hat{\beta}-x_{ij}\hat{t_{j}})}^{2}$ (6)

where $\hat{t_{j}}=\arg\min_{t_{j}} \sum_{i=1}^{N} {(y_{i}-\beta_{0}-x_{i}^{T}\hat{\beta}-x_{ij}\hat{t_{j}})}^{2}$.

The strategy of the ABESS algorithm was to use the most useful features in the C set which had high $\omega_{j}$ values to replace the most useless features in the S set which had low $\varphi_{j}$ values (Figure 1B). The size of replacement is defined as R. So the most useful features in the C set was shown as equation 7 and the most useless features in S set were shown as equation 8.

$C_{R}=\{j\in C:\sum_{i\in C} I(\omega_{j}\leq\omega_{i})\leq R\}$ (7)

$S_{R}=\{j\in C:\sum_{i\in S} I(\varphi_{j}\geq\varphi_{i})\leq R\}$ (8)

So the new feature set after the exchange is :

$\tilde{S}=(S\backslash S_{R})\cup C_{R}$ (9)

When evaluating whether $\tilde{S}$ is better than $S$, the least squares estimate is shown in equation (10).

$\tilde{\beta}=\arg\min_{\beta_{\tilde{U}=0}} \sum_{i=1}^{N} {(y_{i}-\beta_{0}-x_{i}^{T}\beta)}^{2}$ (10)

In equation 10, $\tilde{U}=\{1,\ldots\ldots,p\}\backslash\tilde{S}$. If the bias of $\tilde{S}$ is less than the bias of $S$ (equation 11), the $\tilde{S}$ would be accepted and become a new $S$ set. ABESS algorithm will perform this kind of splicing until the bias doesn't decrease any more or reach the max splicing round.

$\sum_{i=1}^{N} {(y_{i}-\tilde{\beta}_{0}-x_{i}^{T}\tilde{\beta})}^{2}+\tau_{S}<\sum_{i=1}^{N} {(y_{i}-\hat{\beta}_{0}-x_{i}^{T}\hat{\beta})}^{2}$ (11)

The ABESS algorithm in this study was performed using the "abess" package in R. And the max splicing round was set as 20; splicing.type =2; tune.type=”gic”.

### Machine learning methods of basic learners

Given that the severity data contained 4 levels (asymptomatic, mild, severe, critical) in the training set, we regarded it as a regression task, in which 4 distinct levels were labeled from one to four and the input data was expression data (gene-pairs). Next, we chose four kinds of machine learning methods to construct basic learners, including elastic regression net (ERN)[9], random forest (RF)[10], support vector machine (SVM)[11] and k-nearest neighbors (KNN)[12].

ERN algorithm first used weighted L1 regular term and L2 regular term together to penalize the feature coefficients and then performed least squares estimate (equation 12)[13]. Therefore, it is an effective way to identify an appropriate α value in the ERN algorithm to prevent overfitting.

$\hat{\beta}=\arg\min_{\beta} \left[ \sum_{i=1}^{N} {(y_{i}-\beta_{0}-x_{i}^{T}\beta)}^{2}+\alpha\sum_{j=1}^{M} \left\| \theta\right\|+(1-\alpha)\sum_{j=1}^{M} \left\| \theta\right\|_{2}^{2} \right]$ (12)

The KNN algorithm estimates the y label or regression value by selecting the k sample points closest to the input sample point[14], while the SVM algorithm first maps the sample points to the high-dimensional space through kernel functions and then performs further classification or regression prediction[15]. Additionally, the RF algorithm is a kind of ensemble model of decision trees to perform classification or regression tasks, as avoids overfitting well[16]. The principles of the KNN, SVM, and RF algorithms have been fully elaborated in many studies[17-19]. The implement of above 4 kinds of algorithms used “mlr3”,” mlr3learners”, “mlr3viz”, “kknn”, “ranger”, “e1071”, “glmnet”, “caret” packages in R.

To obtain effective models based on these 4 algorithms, we performed 10-fold cross validation and grid search in the training set using Mean Squared Error (MSE)[20] value firstly. The parameters of ERN models were: α=0,0.2,0.4,0.6,0.8 or 1.0; the parameters of RF models were: ntree=200, 500, 800 or 1200, depth=3,5,10 or 20; the parameters of SVM models were: C=1,10,100 or 1000, kernel= “linear”, “radial” or “polynomial”; the parameters of KNN models were: k=3,5,7 or 9, kernel= “optimal”, “rank”, “rectangular” or “triangular”. Finally, we constructed 50 (6+4×4+4×3+4×4) basic learners in training set based on these parameters.

### The construction of AGAE score using a genetic algorithm in the training set.

The 50 basic learners were labeled as m1-m50 sorted by MSE value from low to high in 10-fold CV before these models were used to construct the ensemble learning model in the in-house data (training set). The methods and parameters of the ensemble learning model are consistent with m1 (ERN model, α=1.0). And we used the stacking strategy[21] to construct the ensemble model, so the input data of the ensemble model is the risk scores of basic learners. To ensure the prediction effect of the ensemble learning model, it is necessary to consider both the accuracy of the models and the diversity of the models in the selection of basic learners. To make this process easy to implement, we performed the genetic algorithm (GA) in the training set to optimize the basic learner combination. GA simulates the process of chromosomal recombination evolution in the natural state and is proven to be suitable for genomics-related optimization problems[22], which consist of the fitness function, selection operator, crossover probability, and mutation probability.

In this task, ensemble learning models with different basic learner combinations can be viewed as different chromosomes. And the genes in chromosomes equal to whether specific basic learners are included. “1” means the basic learner is included in the ensemble model and “0” means not. We hope to find the optimal basic learner combination through the evolution of chromosomes. The fitness function was used to evaluate whether a chromosome is good or not as shown in equation 13, which was calculated in the training set using the Spearman coefficient between the predicted value and true severity value.

$f\left( x_{i} \right)=\left\{ \begin{aligned} 1, if no genes exist in chromosome x_{i} \\ 1-{Cor}_{Spearman}(y,\hat{y}), if chromosome x_{i} contains solid gene(s) \end{aligned} \right.$ (13)

And we used the elitism strategy, i.e. keeping the best chromosome unchanged in each round of evolution. Based on initial exploration, we used the roulette strategy to select the chromosome pairs that need to be crossed in a new round of evolution through the selection operator (the selected chromosomes are determined by generating uniformly distributed random number S ($0\leq S\leq1$) and its position in selection operator).

${Selection\_operator}_{i}=\sum_{j=1}^{i} \frac{f(x_{i})}{\sum_{n=1}^{N_{\mathrm{chromosome}}} f(x_{n})}$ (14)

Then, the determination of intersection sites is performed by generating random integer $I$ ($1\leq I\leq50$). After a round of chromosome evolution is completed, each chromosome has a probability M of mutation.

$M=\frac{1}{N_{\mathrm{chromosome}}}$ (15)

And if a chromosome is selected to mutate, N_mg_ genes (random selected) in the mutated chromosome will change. In this task, we set N_mg_=10, N_chromosome_ =50, N_round_ =20.

Then based on the selected basic learner combination, the ensemble model AGAE score would be constructed using the training set.

### The evaluation of the AGAE score

To evaluate the potential robustness and effectiveness of the AGAE score directly, we compared the AGAE scores among the different severity groups using the t-test in the internal test set and 4 external test sets. To further evaluate the AGAE score, we drew the receiver operating characteristic (ROC) curves[23] and then calculated the area under the curves (ROC-AUCs). Because the ROC curve can only be performed in the binary classification tasks, some severity groups in certain cohorts were combined to construct binary groups, and the AGAE score was used as the threshold to draw ROC curves. In the in-house cohort, the "Asymptomatic" group and “Mild” groups were regarded as a group in a good severity state and the "Severe” and “Critical” groups were regarded as a group in a bad severity state. In the GSE155454 cohort, the "severity 0" group and "severity 1" group were regarded as a group in a good severity state and the “severity 2” group was regarded as a group in a bad severity state. The ROC curves were drawn by using the "pROC” package in R and tested by a 300-turn permutation test[24]. Thereafter, we also compared the AGAE score with 5 baseline models, 4 of which were the basic learners in AGAE score, and the last one was the m6A score, which was established by Qiu et al.[25] to predict the degree of severity of COVID-19 patients based on m6A process-related key genes.

Then, to verify whether the gene-pairing method can reduce the batch effect, the gene-pairing method was compared with the ComBat function in the "sva" R package[26]. Principle component analysis (PCA) was used to display feature distribution before or after the usage of the gene-pairing method and ComBat function, respectively. To further verify whether the ABESS algorithm, gene-pairing method, and genetic algorithm can improve the accuracy of the AGAE score, we performed ablation experiments of these three parts. As for the ABESS algorithm, we used the traditional LASSO method to replace it and re-construct the AGAE score. As for the gene-pairing method, we also constructed a new AGAE score based on gene features, not paired. And as for the genetic algorithm, we used all 50 basic learners to construct the ensemble model without a genetic algorithm. The ROC-AUCs in ablation experiments were compared with the original AGAE scores by paired t-test in 5 independent test sets.

In addition, to explore the relationship between AGAE score and clinical characteristics in COVID-19, we selected the GSE157103 cohort to do this which contained many different types of clinical information. We used a t-test to compare the AGAE score in the different discrete clinical variables, including “Gender” and “Mechanical Ventilation”. Thereafter, we used Pearson correlation coefficient to analyze the relationship between the AGAE score and continuous clinical variables, including “Ventilator-free days”, “APACHE-II Score”[27], “SOFA Score”[28], “Age”, “ferritin (ng/ml)”, “ddimer (mg/l_feu)” and “crp (mg/l)”.

SOFA is an acronym for the Sequential Organ Failure Assessment[29]. The SOFA score is based on six different scores, one each for the respiratory, cardiovascular, hepatic, coagulation, renal, and neurological systems. Each of these is rated from 0 to 4 based on objective measurements, with higher scores indicating more severe dysfunction or failure. The total SOFA score can therefore range from 0 (normal) to 24 (high degree of organ dysfunction/failure). The SOFA score can be used to predict the likelihood of mortality in ICU patients. APACHE II (Acute Physiology and Chronic Health Evaluation II) is a patient emergency classification system widely used in ICUs. It's applied within 24 hours of admission of a patient to an ICU. The score is composed of 12 physiological variables and two additional variables related to previous health status. D-dimer is a protein fragment that is produced when a blood clot dissolves in the body. It is often used as a clinical test to help determine if a person might have a blood clotting disorder[30].

Then, we divided the training set into a low AGAE score group and a high AGAE score group. The low AGAE score group was defined as the patients whose AGAE score was less than the median value in the training set. To investigate the possible immune infiltration difference between low and high AGAE score groups, Single-sample Gene Set Enrichment Analysis (ssGSEA)[31] was used. ssGSEA is a deconvolution algorithm based on the bulk gene expression profiles, which can accurately quantify the content of immune cells and predict the degree of immune functions in the cancer tissues. In this process, we used the limma, GSEABase, ggpubr and reshape2 packages in R.

And to verify whether the AGAE score can provide information about key biological processes, Gene set variation analysis (GSVA)[32] was used to study the potential degree of enrichment of the Kyoto Encyclopedia of Genes and Genomes (KEGG) pathways between low and high AGAE score group in the training set. The limma R-package was then used to find differentially-enriched pathways, and the top 20 pathways or terms of false discovery rate (FDR) value with statistically significant fold change (FC) difference (|log_2_FC|>1 and FDR<0.05) were further visualized. The above analyses were carried out using the "GSVA" and "pheatmap" packages in R.

At last, to make AGAE score can be easily used by other researchers and clinicians, we deployed it as a user-friendly web-tool (https://kwkxbioinfor.shinyapps.io/COVID19/) using the "shiny” package in R.

1. Carapito, Raphael, Richard Li, Julie Helms, Christine Carapito, Sharvari Gujja, V. Rolli, R. Guimaraes, et al. 2022. “Identification of driver genes for critical forms of COVID-19 in a deeply phenotyped young patient cohort.” *Sci Transl Med* 14: eabj7521. <https://doi.org/10.1126/scitranslmed.abj7521>

2. Masood, Kiran Iqbal, Maliha Yameen, Javeria Ashraf, Saba Shahid, Syed Faisal Mahmood, Asghar Nasir, Nosheen Nasir, et al. 2021. “Upregulated type I interferon responses in asymptomatic COVID-19 infection are associated with improved clinical outcome.” *Sci Rep* 11: 22958. <https://doi.org/10.1038/s41598-021-02489-4>

3. Overmyer, Katherine A., Evgenia Shishkova, Ian J. Miller, Joseph Balnis, Matthew N. Bernstein, Trenton M. Peters-Clarke, Jesse G. Meyer, et al. 2021. “Large-Scale Multi-omic Analysis of COVID-19 Severity.” *Cell Syst* 12: 23-40.e27. <https://doi.org/10.1016/j.cels.2020.10.003>

4. Wu, Peng, Dongsheng Chen, Wencheng Ding, Ping Wu, Hongyan Hou, Yong Bai, Yuwen Zhou, et al. 2021. “The trans-omics landscape of COVID-19.” *Nat Commun* 12: 4543. <https://doi.org/10.1038/s41467-021-24482-1>

5. Pan, Bei, Yanzhe Yue, Wenbo Ding, Li Sun, Mu Xu, Shukui Wang. 2023. “A novel prognostic signatures based on metastasis- and immune-related gene pairs for colorectal cancer.” *Front Immunol* 14: 1161382. <https://doi.org/10.3389/fimmu.2023.1161382>

6. Zhu, Ssujie, Weikaixin Kong, Jie Zhu, Liting Huang, Shixin Wang, Suzhen Bi, Zhengwei Xie. 2022. “The genetic algorithm-aided three-stage ensemble learning method identified a robust survival risk score in patients with glioma.” *Brief Bioinform* 23: <https://doi.org/10.1093/bib/bbac344>

7. Zhu, Junxian, Canhong Wen, Jin Zhu, Heping Zhang, Xueqin Wang. 2020. “A polynomial algorithm for best-subset selection problem.” *Proceedings of the National Academy of Sciences* 117: 33117-33123. https://doi.org/10.1073/pnas.2014241117

8. Reid, Stephen, Robert Tibshirani, Jerome Friedman. 2016. “A study of error variance estimation in lasso regression.” *Statistica Sinica* 35-67.

9. Hans, Chris. 2011. “Elastic net regression modeling with the orthant normal prior.” *Journal of the American Statistical Association* 106: 1383-1393.

10. Qi, Yanjun. 2012. Random forest for bioinformatics. *Ensemble machine learning* Springer, 307-323.

11. Byvatov, Evgeny, Gisbert Schneider. 2003. “Support vector machine applications in bioinformatics.” *Applied bioinformatics* 2: 67-77.

12. Yao, Zizhen, Walter L. Ruzzo. 2006. A regression-based K nearest neighbor algorithm for gene function prediction from heterogeneous data;7:1-11. BMC Bioinformatics. 7 Suppl 1(Suppl 1):S11. https://doi.org/10.1186/1471-2105-7-S1-S11

13. Ogutu, Joseph O., Torben Schulz-Streeck, Hans-Peter Piepho. 2012. Genomic selection using regularized linear regression models: ridge regression, lasso, elastic net and their extensions; BMC Proceedings. 6 Suppl 2(Suppl 2):S10. https://doi.org/10.1186/1753-6561-6-S2-S10

14. Song, Yunsheng, Jiye Liang, Jing Lu, Xingwang Zhao. 2017. “An efficient instance selection algorithm for k nearest neighbor regression.” *Neurocomputing* 251: 26-34.

15. Balabin, Roman M., Ekaterina I. Lomakina. 2011. “Support vector machine regression (LS-SVM)—an alternative to artificial neural networks (ANNs) for the analysis of quantum chemistry data?” *Physical Chemistry Chemical Physics* 13: 11710-11718. https://doi.org/10.1039/c1cp00051a

16. Segal, Mark R. 2004. “Machine learning benchmarks and random forest regression.”

17. Kramer, Oliver. 2011. “Unsupervised K-nearest neighbor regression.” *arXiv preprint arXiv:1107.3600*,

18. Sun, Bing-Yu, Zhi-Hua Zhu, Jiuyong Li, Bin Linghu. 2010. “Combined feature selection and cancer prognosis using support vector machine regression.” *IEEE/ACM transactions on computational biology and bioinformatics* 8: 1671-1677. https://doi.org/10.1109/TCBB.2010.119.

19. Svetnik, Vladimir, Andy Liaw, Christopher Tong, J. Christopher Culberson, Robert P. Sheridan, Bradley P. Feuston. 2003. “Random forest: a classification and regression tool for compound classification and QSAR modeling.” *Journal of chemical information and computer sciences* 43: 1947-1958. https://doi.org/10.1021/ci034160g

20. Wang, Zhou, Alan C. Bovik. 2009. “Mean squared error: Love it or leave it? A new look at signal fidelity measures.” *IEEE signal processing magazine* 26: 98-117.

21. Yi, Hai-Cheng, Zhu-Hong You, Mei-Neng Wang, Zhen-Hao Guo, Yan-Bin Wang, Ji-Ren Zhou. 2020. “RPI-SE: a stacking ensemble learning framework for ncRNA-protein interactions prediction using sequence information.” *BMC bioinformatics* 21: 1-10. https://doi.org/10.1186/s12859-020-3406-0

22. Mirjalili, Seyedali. 2019. Genetic algorithm. *Evolutionary algorithms and neural networks* Springer, 43-55.

23. Vining, DavidJ, Gregory W. Gladish. 1992. “Receiver operating characteristic curves: a basic understanding.” *Radiographics* 12: 1147-1154.

24. Venkatraman, E. S. 2000. “A permutation test to compare receiver operating characteristic curves.” *Biometrics* 56: 1134-1138. https://doi.org/10.1111/j.0006-341x.2000.01134.x

25. Qiu, Xiangmin, Xiaoliang Hua, Qianyin Li, Qin Zhou, Juan Chen. 2021. “m(6)A Regulator-Mediated Methylation Modification Patterns and Characteristics of Immunity in Blood Leukocytes of COVID-19 Patients.” *Front Immunol* 12: 774776. <https://doi.org/10.3389/fimmu.2021.774776>

26. Leek, Jeffrey T., W. Evan Johnson, Hilary S. Parker, Andrew E. Jaffe, John D. Storey. 2012. “The sva package for removing batch effects and other unwanted variation in high-throughput experiments.” *Bioinformatics* 28: 882-883. https://doi.org/10.1093/bioinformatics/bts034

27. Knaus, William A., Elizabeth A. Draper, Douglas P. Wagner, Jack E. Zimmerman. 1985. “APACHE II: a severity of disease classification system.” *Critical care medicine* 13: 818-829.

28. Lambden, Simon, Pierre Francois Laterre, Mitchell M. Levy, Bruno Francois. 2019. “The SOFA score—development, utility and challenges of accurate assessment in clinical trials.” *Critical Care* 23: 1-9. https://doi.org/10.1186/s13054-019-2663-7

29. Vincent, J. L., Rui Moreno, Jukka Takala, Sheila Willatts, Arnaldo De Mendonça, Hajo Bruining, C. K. Reinhart, PeterM Suter, Lambertius G. Thijs. 1996. 'The SOFA (Sepsis-related Organ Failure Assessment) score to describe organ dysfunction/failure: On behalf of the Working Group on Sepsis-Related Problems of the European Society of Intensive Care Medicine (see contributors to the project in the appendix)', Springer-Verlag.

30. Soheir, S. Adam, S. Key Nigel, S. Greenberg Charles. 2009. “D-dimer antigen: current concepts and future prospects.” *Blood* 113: 2878-2887. https://doi.org/10.1182/blood-2008-06-165845

31. Barbie, David A., Pablo Tamayo, Jesse S. Boehm, So Young Kim, Susan E. Moody, Ian F. Dunn, Anna C. Schinzel, et al. 2009. “Systematic RNA interference reveals that oncogenic KRAS-driven cancers require TBK1.” *Nature* 462: 108-112. <https://doi.org/10.1038/nature08460>

32. Hänzelmann, Sonja, Robert Castelo, Justin Guinney. 2013. “GSVA: gene set variation analysis for microarray and RNA-seq data.” *BMC bioinformatics* 14: 1-15. https://doi.org/10.1186/1471-2105-14-7


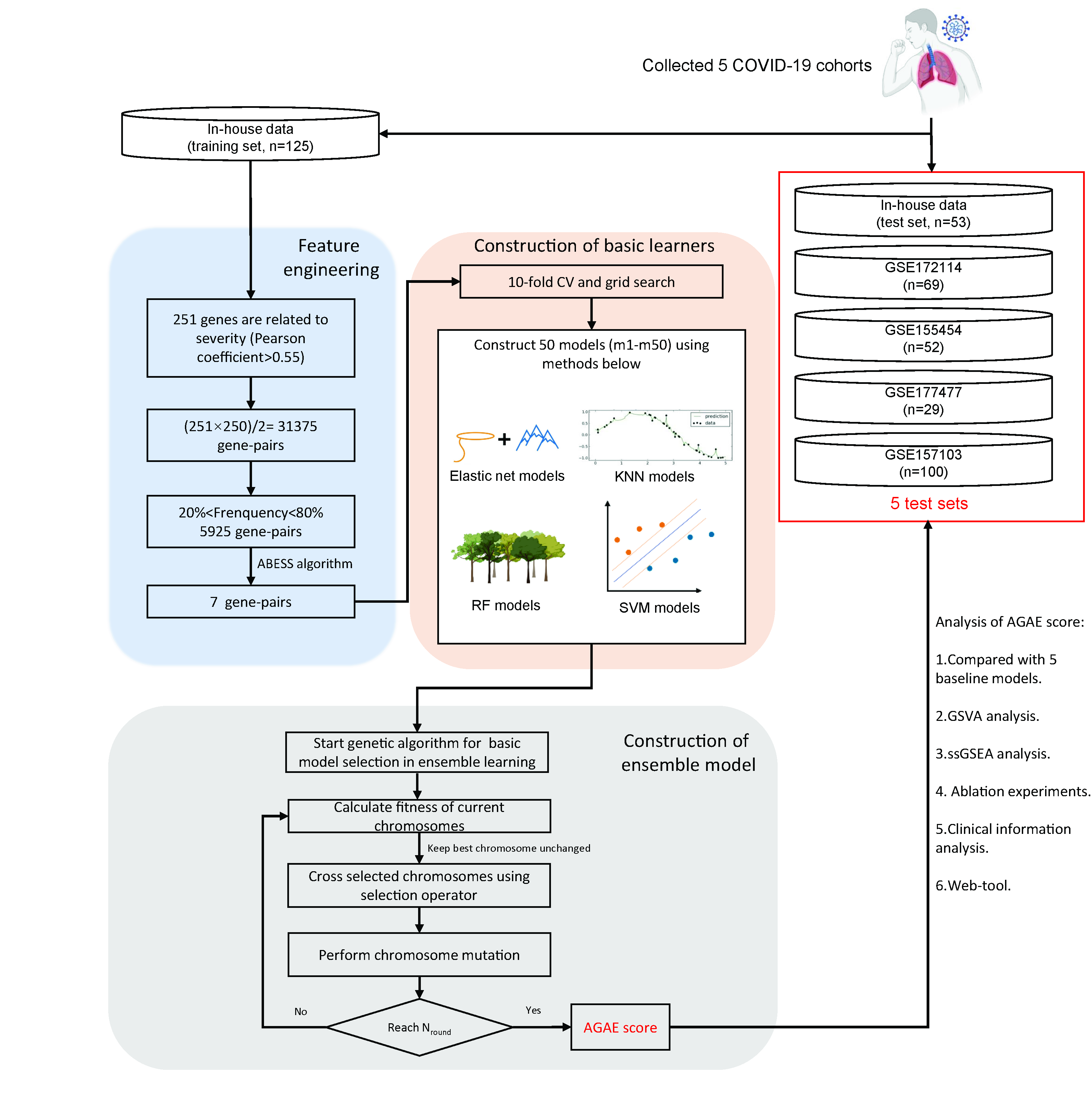


Supplementary Figure 1. The workflow of AGAE score.


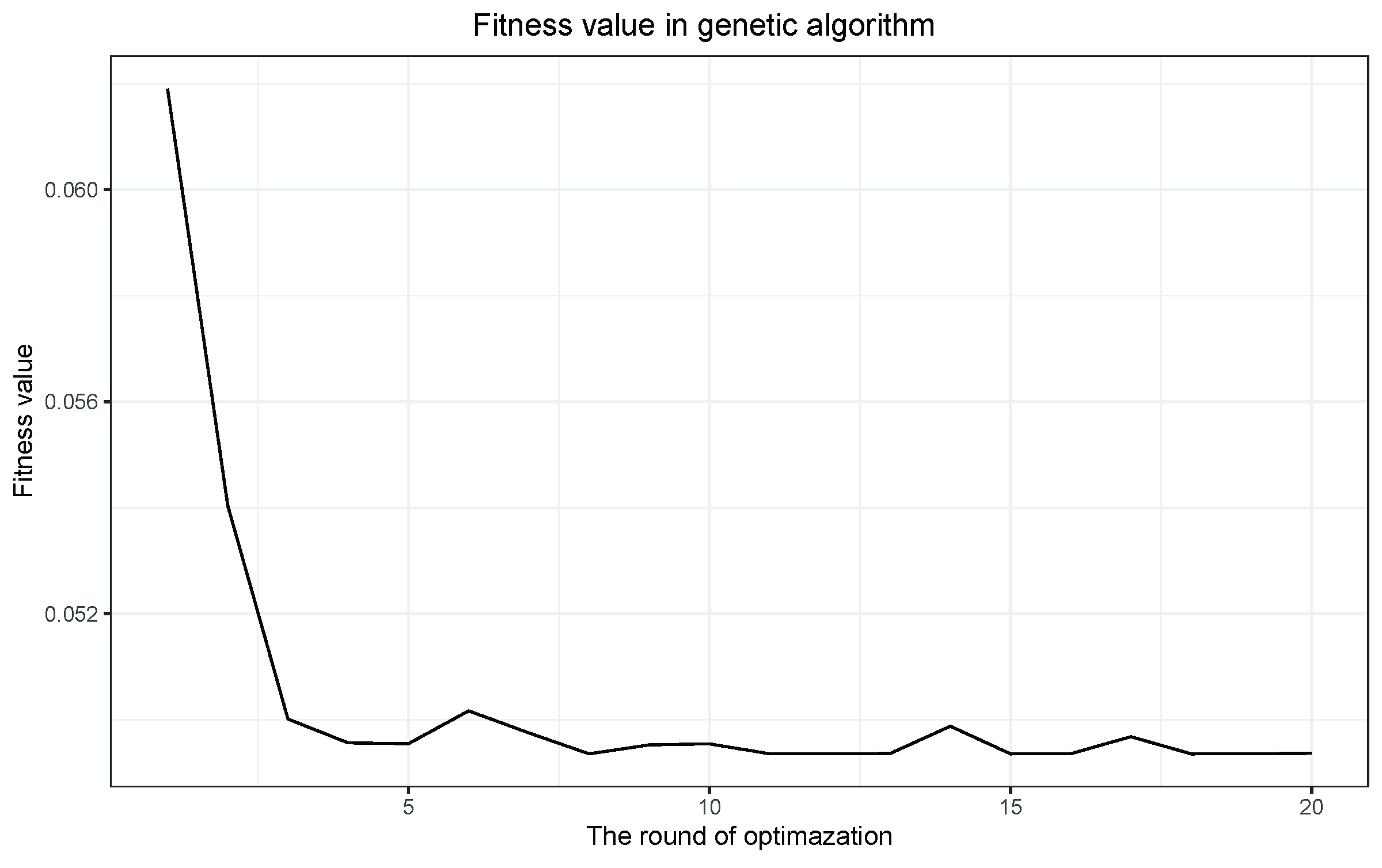


Supplementary Figure 2. The change of fitness value in the genetic algorithm.


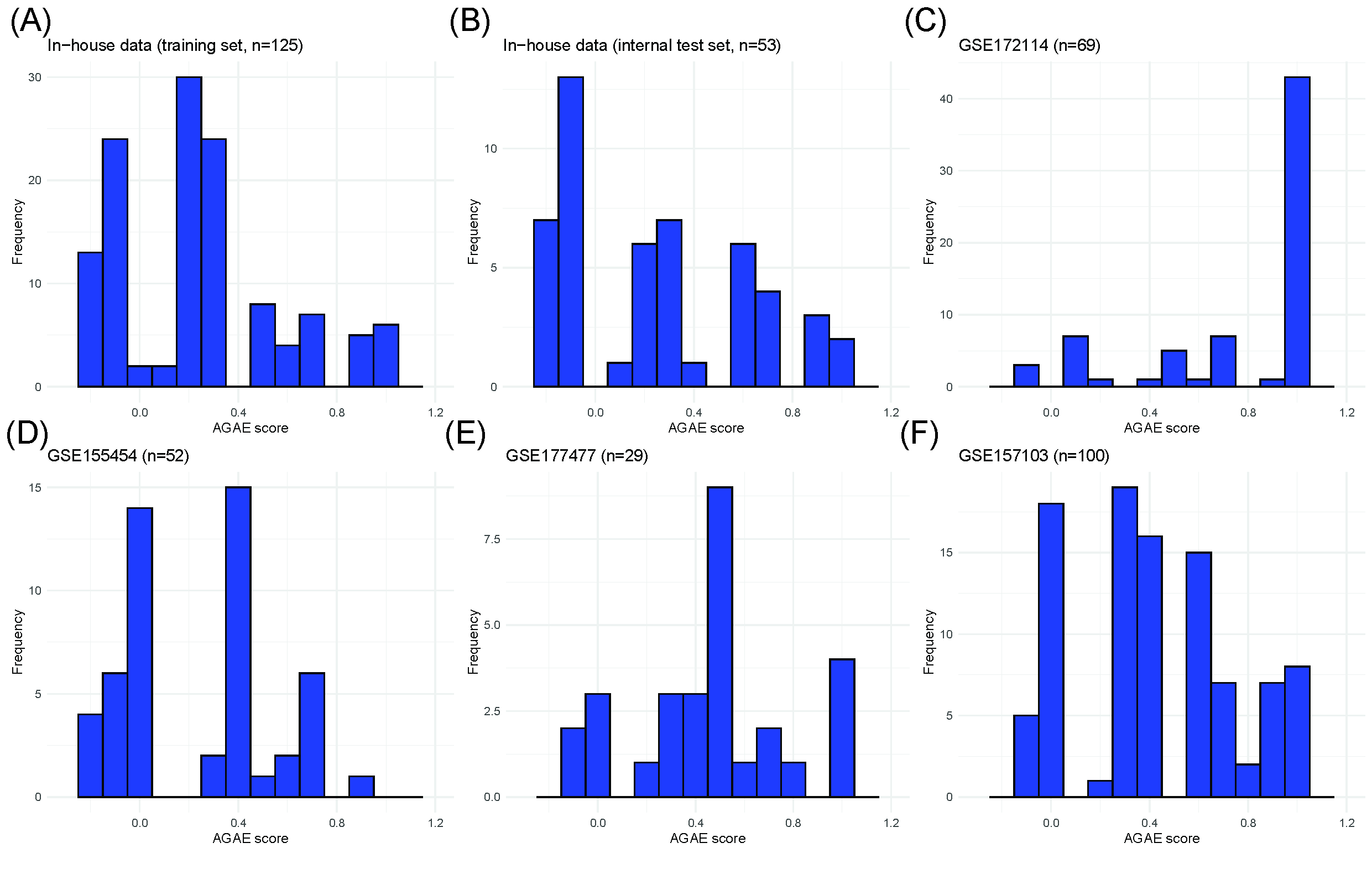


Supplementary Figure 3. The distribution of AGAE score in the training cohort and 5 test cohorts. (A) The training set. (B) Internal test set. (C) GSE172114 cohort. (D) GSE155454 cohort. (E) GSE177477cohort. (F) GSE157103 cohort.


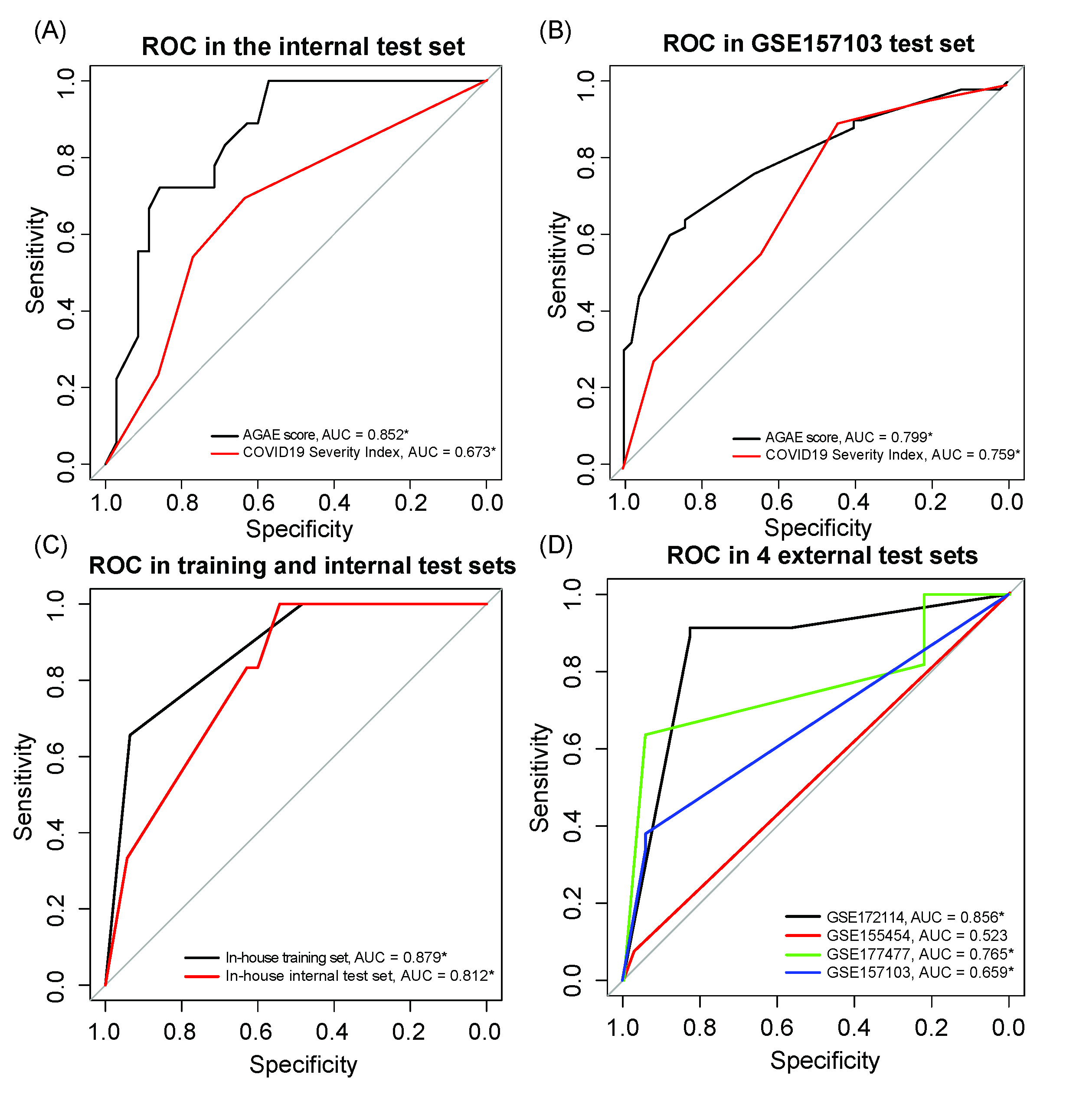


Supplementary Figure 4. (A-B) ROC curves of AGAE score and COVID19 Severity Index in the internal test cohort (in-house) and GSE157103 cohort. (A) The internal test cohort. (B) GSE157103 cohort. (C-D) ROC curves of AGAE score without gene-pairing method in the training set and 5 test sets (300-turn permutation test). (C) The training set and test set (in-house data). (D) 4 external test sets. The samples were divided with different AGAE score thresholds to draw the ROC curves of severity data.


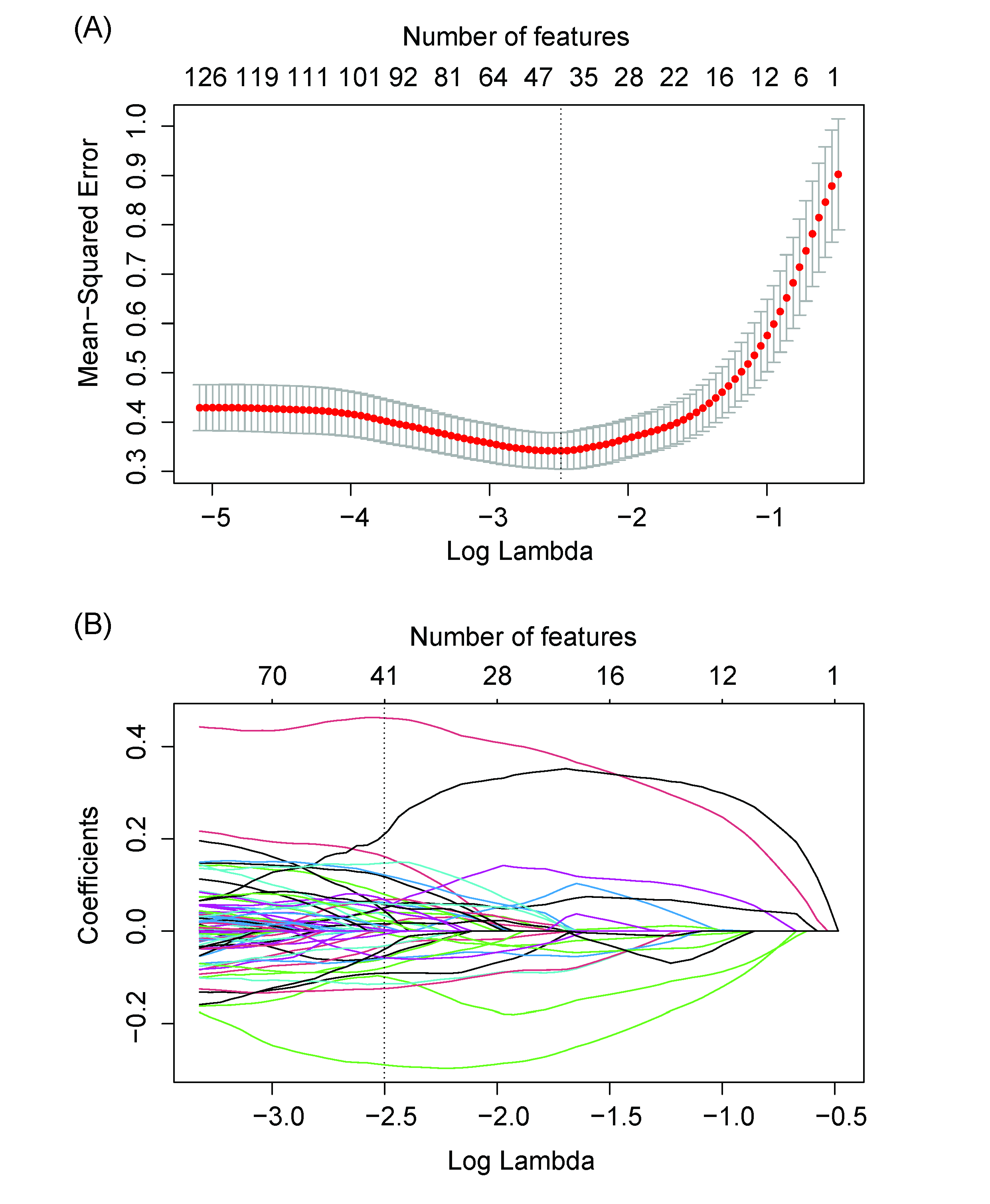


Supplementary Figure 5. The selection of survival predictive gene-pairs using LASSO regression in the in-house data (training set). (A) The mean square error in cross-validation (CV) as a function of the penalty coefficient lambda. The dotted line shows the lambda value of 0.003 at the minimum partial likelihood deviance level, suggesting 41 gene-pairs as optimal predictive features. Standard errors are calculated over 1000 CV rounds. (B) The coefficients of the 41 gene-pairs are the function of the penalty coefficient (lambda).


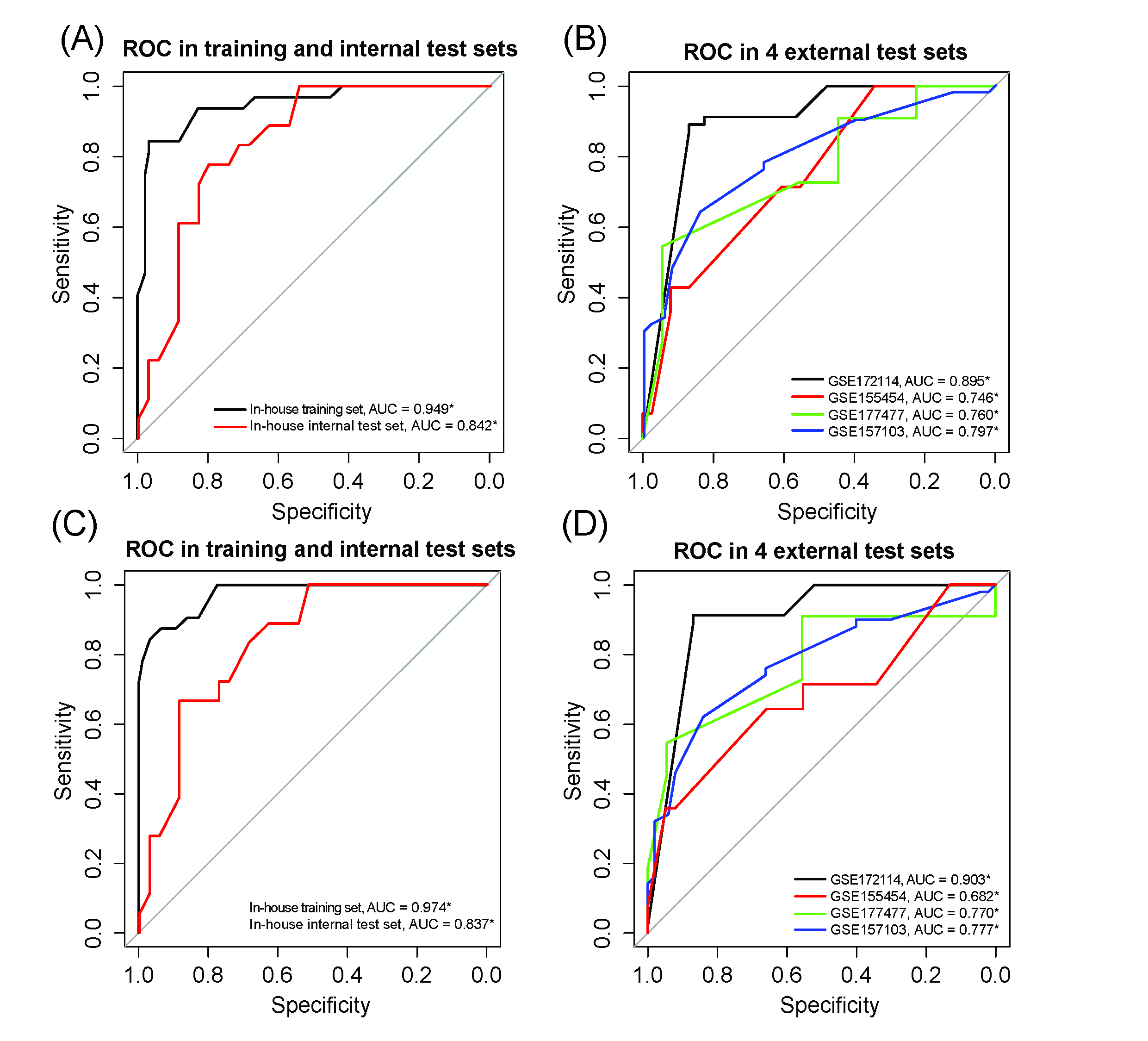


Supplementary Figure 6. (A-B) ROC curves of AGAE score without ABESS algorithm in the training set and 5 test sets (300-turn permutation test). (A) The training set and test set (in-house data). (B) 4 external test sets. The samples were divided with different AGAE score thresholds to draw the ROC curves of severity data. (C-D) ROC curves of AGAE score without genetic algorithm in the training set and 5 test sets (300-turn permutation test). (C) The training set and test set (in-house data). (D) 4 external test sets. The samples were divided with different AGAE score thresholds to draw the ROC curves of severity data.


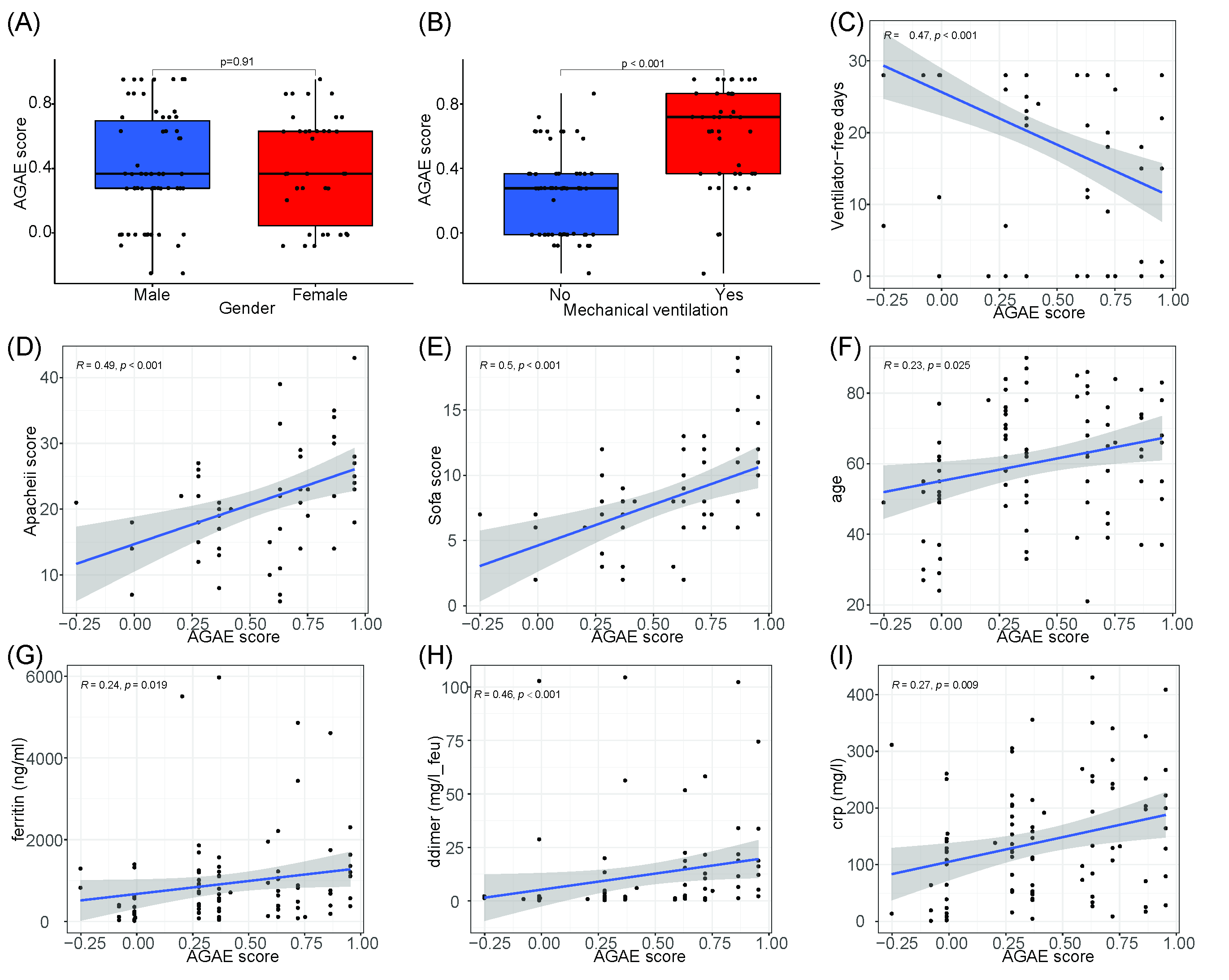


Supplementary Figure 7. The association between AGAE score and different clinical information in the GSE157103 cohort. (A-B) Wilcoxon test. (C-I) Pearson correlation coefficients.


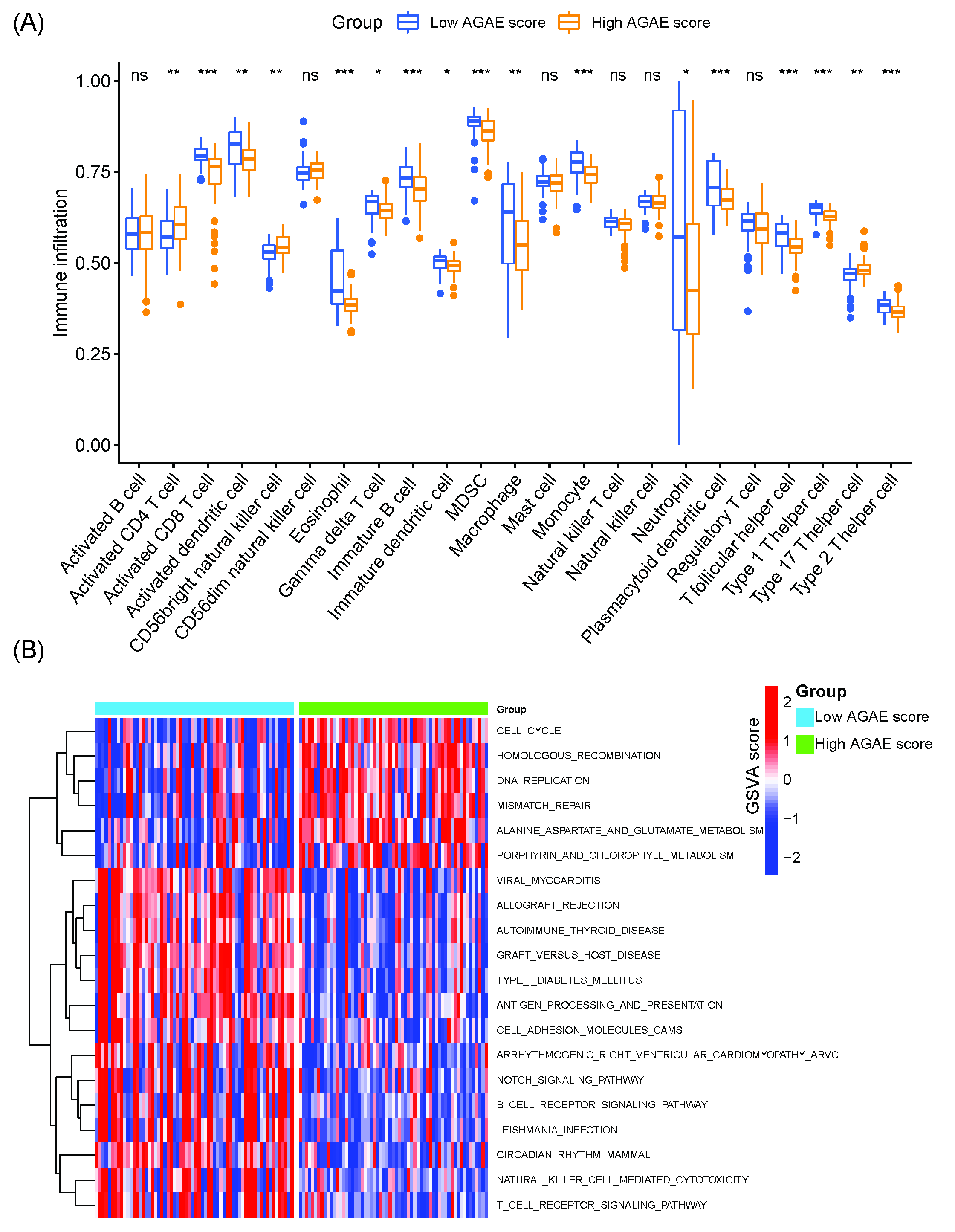


Supplementary Figure 8. (A) Immune infiltration analysis of immune cells in the in-house data (training set) using ssGSEA using Wilcoxon test. **p* <0.05; ***p* <0.01; ****p* <0.001. (B) The enriched KEGG pathways in the GSVA analysis using the AGAE score between the low AGAE score group and high AGAE score group in the in-house data (training set).


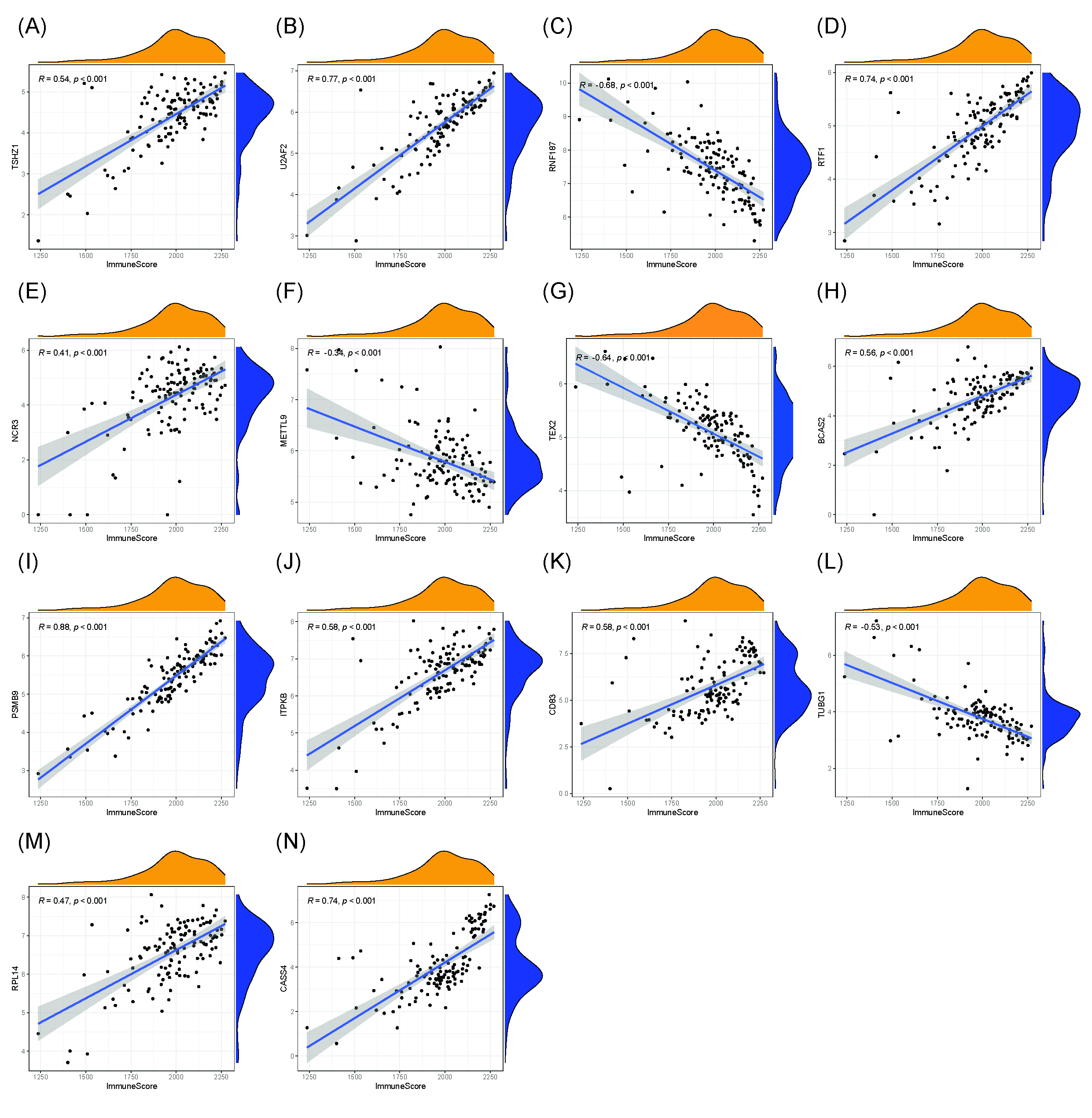


Supplementary Figure 9. Correlation analysis between 14 AGAE-score-gene expressions and ImmuneScore (Spearman correlation coefficient) in the training set. ImmuneScore is obtained by ESTIMATE algorithm and can represent the content of immune cells. The gene expression values are TPM cormat. (A) TSHZ1. (B) U2AF2. (C) RNF187. (D) RTF1. (E) NCR3. (F) METTL9. (G) TEX2. (H) BCAS2. (I) PSMB9. (J) ITPKB. (K) CD83. (L) TUBG1. (M) RPL14. (N) CASS4.
